# A Case of a Large Malignant Pericardial Effusion on Computed Tomography Without Electrocardiographic Gating

**DOI:** 10.7759/cureus.34176

**Published:** 2023-01-25

**Authors:** Rui Flores, Fernando Mané, Inês Conde, Vítor Hugo Pereira, Pedro Azevedo

**Affiliations:** 1 Cardiology, Hospital de Braga, Braga, PRT

**Keywords:** positron emission tomography computed tomography, computed tomography, emergency echocardiography, pulmonary adenocarcinoma, pericardial effusion

## Abstract

A 67-year-old female was admitted due to dyspnea. A computed tomography (CT) disclosed a suspicious pulmonary mass and a pericardial effusion. A transthoracic echocardiogram confirmed a large-volume circumferential pericardial effusion. A pericardiocentesis was performed, and the cytological and histochemical studies later confirmed the diagnosis of pulmonary adenocarcinoma. This case report highlights the casualty of having found a cardiac tamponade through a CT not synchronized with an electrocardiogram.

## Introduction

Malignant pericardial effusions are frequent among patients with cancer and can lead to cardiac tamponade and death [1-3]. Transthoracic echocardiography is the diagnostic gold standard and helps in therapeutic pericardiocentesis [1]. Emerging therapies, such as pericardial injection of sclerosing agents, may change the course of the disease [4].

We report the case of a patient who presented with a symptomatic malignant pericardial effusion requiring urgent pericardiocentesis. The steps that led to the diagnosis emphasize the importance of fortuitous findings in other imaging tests.

## Case presentation

**Figure 1:**
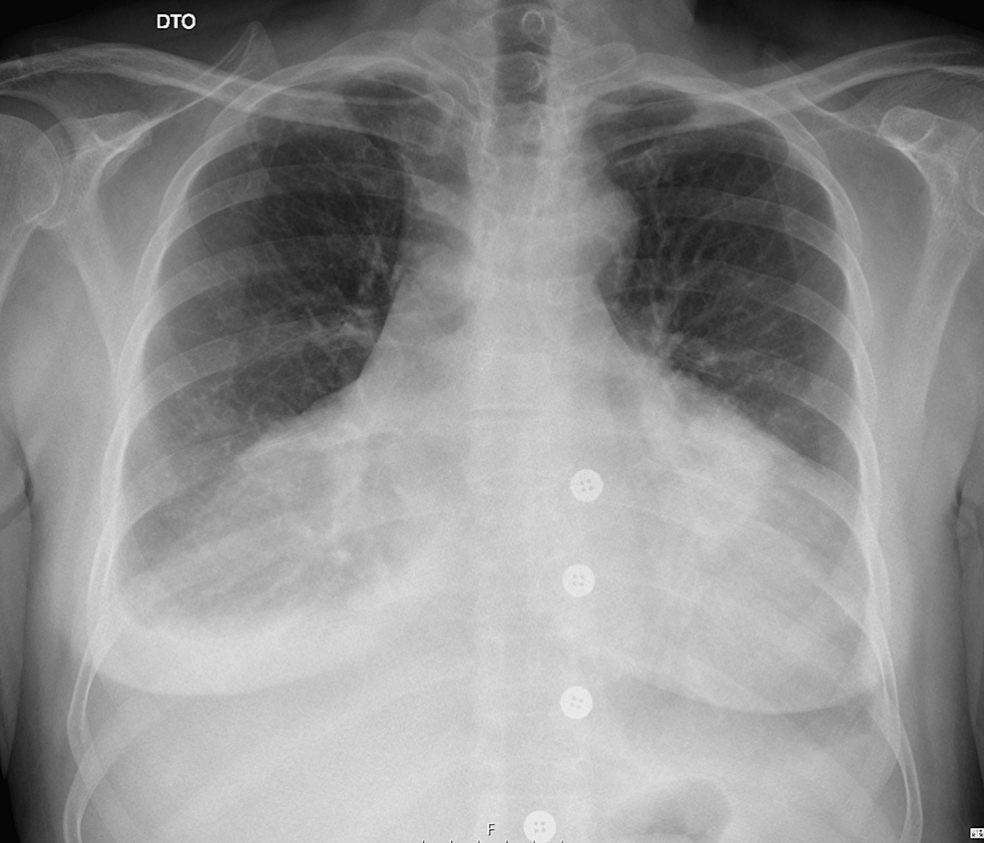
CXR highlighting the patient’s exuberant cardiomegaly. CXR: chest X-ray

A 67-year-old female was admitted to the emergency department due to dyspnea of acute onset. Her past medical history included allergic rhinitis, and she only took bilastine occasionally. She referred a week-old history of progressive worsening dyspnea associated with chest pain and dry cough. At admission, the patient presented tachycardia (heart rate of 120 beats per minute), normal blood pressure (140/80 mmHg), elevated respiratory rate (32 cycles per minute), and normal peripheral oxygen saturation (98% in room air). Pulmonary auscultation revealed diminished respiration sounds in both lung basis. Electrocardiogram showed sinus tachycardia. Chest X-ray (CXR) revealed an enlarged cardiac silhouette and effacement of the lower thirds of both lungs suggestive of pleural effusion (Figure 1).

**Figure 2:**
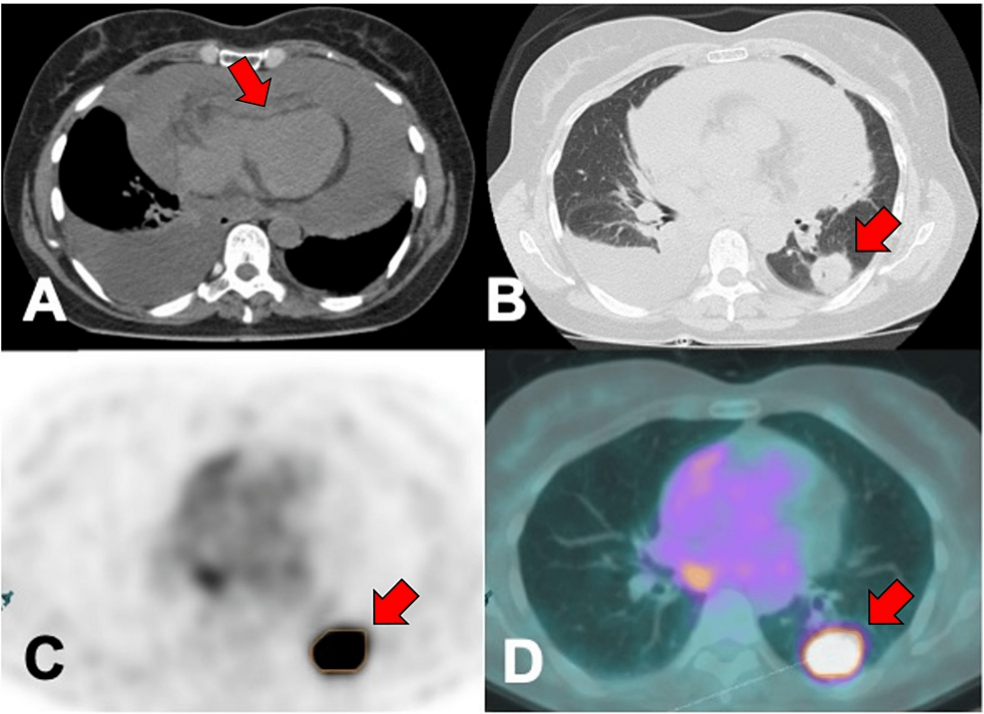
Imaging modalities in the evaluation of pericardial effusion. A: Chest CT scan showing a large-volume pericardial effusion with signs of right ventricular compression (arrow indicating the compressed right ventricle). B: Chest CT scan showing a suspicious lung lesion of nodular morphology with a necrotic center in the left lung (arrow). C and D: PET showing increased FDG uptake by the lung lesion (arrow). CT: computed tomography, PET: positron emission tomography, FDG: fluorodeoxyglucose

The patient underwent computed tomography (CT) scan to clarify these findings, which revealed asymmetrical bilateral pleural effusion and a large pericardial effusion with a diameter of approximately 42 mm causing compression of the right ventricle and dilation of the inferior vena cava (Figure 2A).

In the lung parenchyma, a nodular lesion with suspicious characteristics was observed (Figure 2B). A transthoracic echocardiogram (Video 1) was performed, which confirmed a large circumferential pericardial effusion with stigmata of echocardiographic tamponade, namely, compression of the right heart chambers, pathological variation of transmitral (~70%) and transtricuspid (~80%) flows, and plethoric inferior vena cava.

**Video 1:** Transthoracic echocardiogram in subcostal view. Large-volume pericardial effusion with collapse of the right ventricle in diastole.

An urgent pericardiocentesis was performed with drainage of about 900 mL of pericardial fluid with a hematic appearance, with immediate relief of symptoms. Given the absence of a diagnosis of neoplasia at this stage, the pericardial drain was removed after three days (total drainage of 2,500 mL). Further cytological and histochemical studies confirmed the diagnosis of pulmonary adenocarcinoma. Programmed death-ligand 1 (PD-L1) expression was 60%-70%. Genetic sequencing by next-generation sequencing (NGS) showed a mutation in the MET gene (c.3082+3A>T). In the meantime, positron emission tomography with fluorodeoxyglucose showed localized uptake in the pulmonary mass and small mediastinal adenopathies (Figure 2C and Figure 2D). A full CT scan and a CT of the brain were performed for staging purposes, excluding metastases. According to the TNM staging system, the patient was classified as cT2a N3 M1a. Due to a lack of authorization for other drugs, the patient was started on pembrolizumab.

This case illustrates the importance of pericardial drainage in the diagnosis of malignant pericardial effusions. The risk of recurrence validates the need for additional treatments, namely, the instillation of sclerosing agents to improve the quality of life of patients.

## Discussion

Malignant noninfectious pericardial effusions are frequent findings, occurring in up to 10% of autopsies of cancer patients [1-3]. Direct metastasis and side effects of chemotherapy and radiotherapy are the most frequently involved mechanisms, although cancer-induced immunosuppression may play a role in infectious effusions [1,4]. Direct lymphatic invasion may also play a role [4]. Cancers with more frequent pericardial involvement include lung, breast, skin, and hematological cancers [1].

Malignant pericardial effusions can be fatal, mostly in cases of pericardial tamponade; however, the symptoms and signs are variable according to the effusion installation time and possible associated pericardial inflammation [1,4]. The accumulation of fluid in the pericardial space leads to an increase in intrapericardial pressure [5]. With progressive accumulation, intrapericardial pressure may exceed intracardiac pressure, and cardiac chambers collapse (most often the right chambers as they subserve a low-pressure system) [5]. Due to ventricular interdependence, bulging of the interventricular septum toward the left ventricle decreases the end-diastolic volume and may compromise cardiac output, leading to hypotension and signs of low cardiac output typical of pericardial tamponade [5]. This interventricular dependence is accentuated by the phases of the respiratory cycle [5]. For instance, with inspiration, the increase in venous blood return increases the pressure in the right chambers, exacerbating the deviation of the interventricular septum and leading to a characteristic pulse waveform (pulsus paradoxus) [5].

Transthoracic echocardiography is the first-line examination for the definitive diagnosis of pericardial effusion and cardiac tamponade, although there are other imaging modalities, namely, magnetic resonance imaging and computed tomography [1]. These two are theoretically capable of diagnosing tamponade, although it requires synchronization with an electrocardiogram for the proper assessment of limitation to ventricular diastolic filling [1,2]. Other examinations, such as electrocardiogram and chest radiography, have low diagnostic sensitivity and specificity for small-volume effusions [5]. More specific echocardiographic findings of cardiac tamponade include the collapse of the right chambers, plethoric inferior vena cava, and exaggerated variation in transmitral, transtricuspid, and left ventricular outflow tract flow [5].

Pericardiocentesis is a procedure that is both diagnostic and therapeutic in most patients, especially when the pericardial effusion represents the first manifestation of oncological disease [1]. In most cases, percutaneous and echocardiogram-guided pericardiocentesis is possible, and cytological analysis of the pericardial fluid can be useful to unravel the underlying oncological diagnosis [4]. As the risk of recurrence is high (up to 60%), it is important to consider other adjuvant therapies to this percutaneous technique, such as the instillation of sclerosing agents, rapid initiation of treatments directed at the neoplasm, pericardiotomy, and a pericardial window [1,4,6]. As the survival of cancer patients has been increasing exponentially, it is increasingly common to find complications resulting from pericardial involvement and/or pericardiocentesis(es), such as constrictive pericarditis [5].

Compared to infectious or non-neoplastic noninfectious causes, the prognosis of malignant pericardial effusions, especially those requiring pericardiocentesis, is significantly worse [5].

We report the case of a previously asymptomatic female who presented with complaints of dyspnea, chest pain and dry cough, and echocardiographic stigmata of cardiac tamponade. Pericardiocentesis performed urgently for symptomatic relief was important for the definitive diagnosis of oncological disease, as well as the quick initiation of chemotherapy. From our center’s experience, pericardiocentesis in malignant scenarios is often the first step in formulating the diagnosis of malignant disease, often with pericardial fluid with a hematic appearance, and whose cytological analysis is extremely important. The instillation of sclerosing agents, such as cisplatin, can have an impact on improving the quality of life of patients; however, as at the time of the removal of the pericardial drain, there was still no formal diagnosis of cancer, the decision was not to apply any pericardial therapy. This case is curious, mainly since the suspicion of tamponade was made using another imaging technique, namely, CT, without any synchronization with an electrocardiogram.

## Conclusions

Malignant pericardial effusion may be the first manifestation of oncological disease, and there are several possible underlying pathological mechanisms that alter the prognosis of the underlying disease. Pericardiocentesis, urgent or elective, can be very useful for the definitive diagnosis. The combination of other imaging tests is essential after clinical suspicion. This case reveals the importance of an in-depth study of pericardial effusions and the importance of valuing fortuitous findings from other imaging tests.
